# Assessing Peer Exposure at a Group Level: The Role of Mild-to-Moderate Symptoms in the Transmission of Mental Health Problems

**DOI:** 10.1155/da/1787378

**Published:** 2025-04-21

**Authors:** Dongyu Liu, Xiyu Wei, Luxia Jia, Sai Huang, Bao Zhang

**Affiliations:** ^1^School of Education, Guangzhou University, Guangzhou, China; ^2^School of Public Health, LKS Faculty of Medicine, The University of Hong Kong, Hong Kong Special Administrative Region, China; ^3^Department of Social Work and Social Administration, The University of Hong Kong, Hong Kong Special Administrative Region, China

## Abstract

**Background:** Preliminary evidence suggests that mental health problems can transmit within adolescent peer social groups. However, prior studies have primarily quantified exposure by counting peers with mental health problems, which cannot precisely reflect the group level density of already affected peers. Moreover, existing evidence predominantly focused on transmission associated with exposure to severe cases, neglecting the more widely prevalent mild-to-moderate cases. Therefore, we explored whether and in what condition exposure to mild-to-moderate cases should be considered along with severe cases in the transmission of mental health problems.

**Methods:** We analyzed data from a mental health monitoring project involving 20 middle schools in Guangdong, China, encompassing all students in 449 classes. The risks of adolescents reporting worse depressive and anxiety symptoms associated with exposure to classmates with severe symptoms or mild-to-severe symptoms were explored through three-level mixed-effect multiple Poisson regression models, adjusted for random effects at classroom and school levels.

**Findings:** Among the 19,058 participants (48.3% girls), 5651 (29.6%) reported depression problems and 6464 (33.9%) reported anxiety problems. Higher risks of adolescents reporting worse depressive and anxiety symptoms were significantly associated with exposure to classmates with any severity of symptoms when considering the percentage of these classmates in the classroom (IRR ranged between 1.01 and 1.02). Conversely, having greater number of classmates with severe symptoms was significantly associated with higher risk of reporting worse symptoms (IRR ranged between 1.03 and 1.09) regardless of proportion of these classmates.

**Interpretation:** Our findings indicated that in group level mental health transmission, the level of exposure should be interpreted with consideration of group density rather than mere number of already affected peers. Moreover, mental health problems can transmit beyond exposure to peers with severe symptoms, thereby facilitating more effective risk screening and prevention of mental health problem transmission in adolescents. This approach is imperative, given the substantial prevalence of mild-to-moderate mental health symptoms among adolescents.

## 1. Introduction

Mental health problems, particularly depression and anxiety, have become a leading burden of disease among adolescents [1] with prevalence rates of 4.4% and 1.4% in 10–14-year-olds, and 5.5% and 3.5% in 15–19-year-olds, respectively [1]. In China, the prevalence of having co-occurring symptoms of depression and anxiety in middle school students has reached 20.6% [2]. In adolescents, peer-related social environments have been shown as crucial for mental health development [3]. Current evidence has emphasized the importance of maintaining good peer connectedness and peer relationship quality in adolescent mental health [4, 5]. Meanwhile, it has been shown that the previously overlooked potential negative impacts of interpersonal relationship on mental health is equally important, as mental health problems are interpersonally transmittable [6, 7].

It has been well-established that mental health problems can transmit between close friends [8–10], roommates [11], spouses [12], as well as intergenerationally [13–15]. Moreover, evidence from institutionally imposed groups in school environment suggested that the transmission of mental health problems exist beyond people with close personal relationships [6, 16]. This evidence further indicated that the transmission of mental health problems is independent from self-selection effect and shared genetic predispositions [17–19]. Group level transmission is particularly relevant for adolescents as they receive substantial influence from the social, emotional, and physical dynamics within a stable group of classmates in school environment [20]. This is especially true in Chinese middle schools, where high academic pressure elevates the risk of mental health problems [21, 22], thereby increasing the likelihood of transmission among classmates.

When considering group level transmission in adolescents, previous studies generally suggested a dose-responsive relationship in which the transmission is associated with level of exposure to peers with mental health problems [6, 16]. This indicated the importance of considering a group level density of peers with mental health symptoms, as an individual is exposed to peers with and without mental health symptoms simultaneously in a group [16]. Nevertheless, previous studies only used the number of peers with mental health problems as indicator of exposure. This counting indicator is insufficient in capturing the combined influence of both already affected peers and healthy peers on group dynamic, thus, cannot precisely reflect group level density of peers with mental health symptoms. This limitation is critical in stable peer groups such as classroom environment, where adolescents are shaped by both direct peer influence and the broader group dynamic formed by all members [20, 23, 24]. Recent studies have employed the group level density estimation to examine the transmission of educational behaviors in secondary students, showing that exposure to a higher proportion of peers engaging in private tutoring increases the likelihood of similar behavior in the ego [25, 26]. However, this approach has not yet been applied to mental health transmission. Advancing the field requires more precise estimations of peer exposure, incorporating group level density, to better understand the dynamics of mental health transmission.

Another important point when considering group level transmission is the inclusion of peers with mild-to-moderate symptoms when estimating level of exposure, as all members equally contribute to group dynamic, which is influential to mental health in adolescents [20, 27]. While current studies generally applied a severe cases-focused approach, it should be noted that there are more widely prevalent cases with mild-to-moderate symptoms [28, 29]. Therefore, it is vital to also consider the exposure to peers with mild-to-moderate symptoms along with severe cases when exploring mental health transmission. While the chance of exposure to peers with severe mental health symptoms is relatively low, being in a group with a high density of peers exhibiting mild-to-moderate symptoms is more probable and may also trigger transmission. Moreover, exploring the role of mild-to-moderate symptoms in the transmission of mental health might facilitate a better understanding of the subtle group-level mechanisms of transmission. Direct interpersonal mechanisms, such as empathy and co-rumination, identified in the context of severe symptoms [7, 8] may not apply to mild-to-moderate symptoms, which often go unnoticed in typical school settings [30]. Instead, individuals with mild-to-moderate symptoms may influence peers through their collective impact on group dynamics [20]. Considering adolescent peer groups as a complex system [31], the tipping point theory indicated that the group dynamics remain stable until the proportion of individuals with such symptoms crosses a threshold [32], potentially triggering the transmission of depressive and anxiety symptoms. Yet, no study of mental health problems transmission to-date has explored the association of exposures to peers with mild-to-moderate symptoms along with severe cases on the ego's mental health outcomes.

Therefore, the present study aimed to utilize data from entire classes in Chinese middle schools to explore the role of mental health problem density and symptom severity in the transmission of depression and anxiety among complete classes of adolescents. We hypothesis that transmission of depression and anxiety is associated with exposures to classmates with any level of symptoms when considering the classroom density of these classmates.

## 2. Materials and Methods

### 2.1. Study Design and Population

This cross-sectional study utilized a clustered-sampling method, with the study population comprising all middle school students (grade 7–9) within available classes of 20 schools in Guangdong province, China. Data were utilized from a school-level student mental health monitoring project from February to April 2023. All students in the participating classes completed the monitoring questionnaire, and all mental health questions were made mandatory, thus, there were no missing data regarding mental health in the dataset. School classes with fewer than 10 students were excluded from analysis to ensure meaningful group level analysis. One student was excluded from the analysis due to being suspended and not belonging to any class. All students in the remaining classes were included when calculating exposure scores, while 1229 students with missing information of sex were removed from statistical modeling. Consequently, a total of 19,058 students from 448 classes were included as participants. The participating schools and parents of the students gave consent for the data to be utilized in research. The study received approval from the ethics committee of the University of Guangzhou (2024080) and followed the Strengthening the Reporting of Observational Studies in Epidemiology reporting guidelines.

The present study utilized classes as the institutionally imposed groups for exposure estimation. Chinese secondary school class allocation primarily follows a stratified randomization approach based on academic ability [33]. This prevents from the potential self-selection effect based on mental health.

### 2.2. Depression and Anxiety

Participants' symptoms of depression and anxiety were assessed through the depression and anxiety sub-scales of the Mental Health Inventory of Middle school students (MMHI-60), respectively [34]. The MMHI-60 was specifically developed for Chinese middle school students, which consists of ten domains of psychosocial adjustments, each comprising six items. Participants rated the extent to which they agree with the statement of each item based on a 5-point Likert scale. The mean score of the six items in each domain, ranging from 1 to 5, represents the severity of mental health symptoms, with higher scores indicating more severe mental health symptoms. Participants with a total score of 2 or higher were classified as having mental health problems. A score of 2–2.99 indicates mild symptoms, a score of 3–3.99 indicates moderate symptoms, and a total score of 4–5 indicates severe symptoms. Having moderate-to-severe symptoms is a recommended cutoff for help-seeking. The MMHI-60 has been widely applied to Chinese middle school students, with good reliability and validity [34–36]. In the current sample, the internal consistency of both subscales for depression (α = 0.87) and anxiety (α = 0.91) reached a good standard.

### 2.3. Demographics

Participants self-reported their biological sex and participants' grade was systematically linked to the classes they belonged to.

### 2.4. Statistics

Data analysis was conducted through R 4.3.3 [37]. Descriptive statistics were first conducted for all variables of interest. Means and standard deviations were calculated for normally distributed continuous variables, while medians and ranges were calculated for non-normally distributed continuous variables. Counts and percentages were calculated for categorical variables.

Exposure to classmate depression and anxiety was calculated in two ways, including the precise number of classmates with depression or anxiety, and the percentage of classmates with these conditions, representing the density of these students in the classroom. The percentage scores were calculated by having the number of classmates with these conditions divided by the total number of classmates. The participant's own symptoms were excluded from these calculations to ensure that calculated scores precisely capture exposure received from classmates. Exposure to classmates with depression and anxiety was calculated separately. To explore the role of mild-to-moderate symptoms in the transmission of depression and anxiety while accounting for the role of severe symptoms, exposure was classified in two means, by severe symptoms (total score 4–5) and mild-to-severe symptoms (total score 2–5). The two exposure indicators were separately utilized as predictors for the ego's mental health in statistical models. Involving already affected individuals with all symptom severities in exposure estimation reflects the real-world scenario, where all contribute to shaping the group dynamic and should be considered collectively [20, 27]. In contrast, the severe-symptom-only exposure indicator represents the conventional approach in mental health transmission studies, which focuses solely on severe cases.

The risks of the ego having severe symptoms and moderate-to-severe symptoms associated with exposure to classmates with mild-to-severe symptoms were first assessed. Students without any symptoms were not involved in this step to prevent the bias caused by these students having one more classmate with symptoms compared to their classmates with symptoms. Then, the risks of the ego having moderate symptoms or mild-to-moderate symptoms associated with exposure to classmates with severe symptoms were assessed to further explore the difference between involving and not involving exposures to classmates with mild-to-moderate symptoms when assessing mental health transmission. Students with severe symptoms were excluded at this stage to prevent the bias caused by these students having one less classmate with severe symptoms compared to their classmates without severe symptoms. Cross-transmission was also explored through having exposure to classmates' depressive symptoms predicting ego's risk of anxiety symptoms, and vice versa.

Three-level mixed-effect multiple Poisson regression models were conducted through the “lme4” package in R to explore the association between exposure to classmates with depression or anxiety and higher risk of the ego reporting these conditions [38]. Though our participants were clustered by institutionally imposed classrooms, potential reflection problems might exist due to common influence at classroom- and school-level, such as teacher's influence and school quality [39]. Therefore, random effects were controlled for at the classroom- and school-level to adjust for potential confounding effects from common external factors in the classroom and school environments. The significance level was set as α = 0.05.

Considering the wide variety of class sizes in our data, sensitivity analyses were conducted for participants in classes of regular class sizes. Based on the most recent release of the Chinese Statistical Bulletin on National Education Development and classifications of Chinese school sizes [40, 41], 16,306 participants from 402 classes (class size of 30–56) were involved in the sensitivity analyses.

## 3. Results

Descriptive statistics of the main outcomes are presented in Table 1. Among the 19,058 participants involved in the analysis, 48.3% were girls and 51.7% were boys. The class size ranged between 13 and 104 students (median = 48). A total of 5651 participants (29.6%) reported having depressive symptoms, in which 404 (2.1%) reported having severe symptoms. A total of 6464 participants (33.9%) reported having anxiety symptoms, in which 664 (3.5%) reported having severe symptoms.

### 3.1. Transmission of Depressive and Anxiety Symptoms

**Figure 1:**
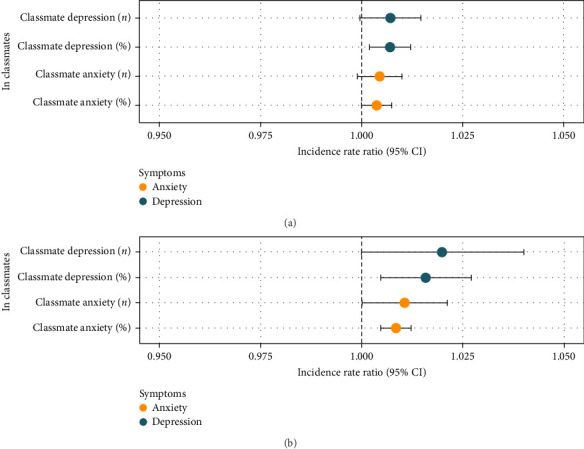
Mental health risks associated with exposure to classmates with mild-to-severe mental health symptoms. In students with mild-to-severe symptoms. Random effects were controlled for at classroom and school levels. (a) Risks of having moderate-to-severe symptoms of depression and anxiety. (b) Risks of having severe symptoms of depression and anxiety.

As shown in Figure 1, while no significant association was found between the precise number of classmates with mild-to-severe symptoms and the higher risk of the ego reporting worse symptoms, the percentage of classmates with mild-to-severe depressive symptoms was significantly associated with the higher risk of reporting moderate-to-severe symptoms (Figure 1a; IRR = 1.01, 95% CI = [1.01, 1.01]) and severe symptoms (Figure 1b; IRR = 1.02, 95% CI = [1.01, 1.03]) of depression. The percentage of classmates with mild-to-severe anxiety symptoms was also significantly associated with higher risks of reporting severe anxiety symptoms (Figure 1b; IRR = 1.01, 95% CI = [1.01, 1.01]).

**Figure 2:**
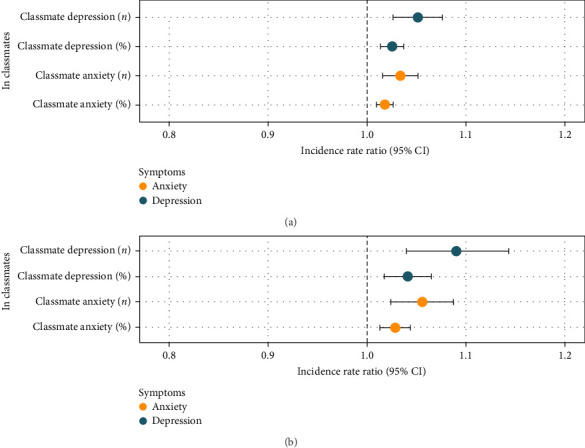
Mental health risks associated with exposure to classmates with severe mental health symptoms. In students without severe symptoms. Random effects were controlled for at classroom and school levels. (a) Risks of having mild-to-moderate symptoms of depression and anxiety. (b) Risks of having moderate symptoms of depression and anxiety.

In comparison, Figure 2 illustrates that students exposed to greater number of classmates with severe depressive and anxiety symptoms were significantly associated with higher risks of having mild-to-moderate depressive symptoms (Figure 2a; IRR = 1.05, 95% CI = [1.03, 1.08]) and anxiety symptoms (Figure 2a; IRR = 1.03, 95% CI = [1.01, 1.05]), as well as having moderate depressive (Figure 2b; IRR = 1.09, 95% CI = [1.04, 1.15]) and anxiety symptoms (Figure 2b; IRR = 1.06, 95% CI = [1.02, 1.09]), respectively. When considering mental health problem density, the results indicated a consistently significant association of the percentage of classmates with severe depressive and anxiety symptoms with higher risks of having mild-to-moderate depressive (Figure 2a; IRR = 1.03, 95% CI = [1.01, 1.04]) and anxiety (Figure 2a; IRR = 1.02, 95% CI = [1.01, 1.03]) symptoms, as well as having moderate depressive (Figure 2b; IRR = 1.04, 95% CI = [1.02, 1.07]) and anxiety (Figure 2b; IRR = 1.03, 95% CI = [1.01, 1.04]) symptoms, respectively.

The results from sensitivity analyses (Supporting Information Figures S1 and S2) indicated that the results are generally stable. When assessing the exposure to already affected classmates with mild-to-severe depressive and anxiety symptoms, the precise number of already affected classmates was not significantly associated with the ego's risk, except for the higher risk of the ego reporting severe depressive symptoms associated with a higher number of classmates with depressive symptoms (IRR = 1.03, 95% CI = [1.01, 1.06]). In contrast, the percentage of already affected classmates with mild-to-severe depressive symptoms was significantly associated with a higher risk of the ego reporting severe (IRR = 1.02, 95% CI = [1.00, 1.03]) and moderate-to-severe depressive symptoms (IRR = 1.01, 95% CI = [1.00, 1.01]). The percentage of already affected classmates with mild-to-severe was significantly associated with a higher risk of the ego reporting severe anxiety symptoms (IRR = 1.01, 95% CI = [1.00, 1.02]). Exposure to already affected classmates with severe depressive and anxiety symptoms was significantly associated with the ego's risk of mild-to-moderate and moderate symptoms regardless of how exposure was estimated (IRR ranged between 1.01 and 1.16).

### 3.2. Cross Transmission of Depressive and Anxiety Symptoms

As shown in Table 2, exposure to a greater number of classmates with severe depressive symptoms was significantly associated with a higher risk of reporting mild-to-severe anxiety problems (IRR = 1.04, 95% CI = [1.02, 1.07]), moderate-to-severe anxiety symptoms (IRR = 1.08, 95% CI = [1.04, 1.12]), and severe anxiety symptoms (IRR = 1.16, 95% CI = [1.12, 1.20]). These associations remained significant when considering mental health problem density, as having a higher percentage of classmates with severe depressive symptoms was significantly associated with a higher risk of mild-to-severe anxiety symptoms (IRR = 1.02, 95% CI = [1.01, 1.03]), moderate-to-severe anxiety symptoms (IRR = 1.04, 95% CI = [1.02, 1.06]), and severe anxiety symptoms (IRR = 1.07, 95% CI = [1.05, 1.09]). Similarly, significant anxiety-to-depression transmission was identified, as a greater number of classmates with severe anxiety symptoms was significantly associated with a higher risk of reporting mild-to-severe depressive symptoms (IRR = 1.03, 95% CI = [1.01, 1.05]), moderate-to-severe depressive symptoms (IRR = 1.05, 95% CI = [1.01, 1.09]), and severe depressive symptoms (IRR = 1.12, 95% CI = [1.07, 1.17]). These associations also remained significant when considering problem density, as having higher percentage of classmates with severe anxiety was significantly associated with higher risk of reporting any depression problems (IRR = 1.02, 95% CI = [1.01, 1.02]), moderate-to-severe depressive symptoms (IRR = 1.03, 95% CI = [1.01, 1.04]), and severe depressive symptoms (IRR = 1.08, 95% CI = [1.02, 1.14]). No cross-transmission was identified for exposure to classmates with mild-to-severe mental health symptoms.

The results from sensitivity analyses (Supporting Information Table S1) indicated that the results were generally stable. Exposure to already affected classmates with severe depressive symptoms was significantly associated with a higher risk of the ego reporting anxiety symptoms regardless of how exposure was estimated (IRR ranged between 1.02 and 1.18). Exposure to already affected classmates with severe anxiety symptoms was significantly associated with a higher risk of the ego reporting depressive symptoms regardless of how exposure was estimated (IRR ranged between 1.02 and 1.21).

## 4. Discussion

Utilizing data from entire classes of 19,058 Chinese middle school students, the present study extends the transmission of mental health problems in adolescent peer groups that the transmission can exist with exposure to peers with any level of mental health symptoms. More importantly, our findings identified an intricate interaction between the group density of peers with mental health symptoms and the symptom severity of these peers. Specifically, higher risks of mental health problems were associated with exposure to already affected classmates irrespective of their symptom severity, but only when considering the density of these classmates in the classroom.

While supporting the transmission of mental health problems in adolescent peer groups, our findings further indicated the importance of incorporating exposure to peers with mild-to-moderate symptoms while considering classroom density of peers with mental health problems when estimating a group level risk of transmission. Specifically, having higher percentage of already affected classmates with any level of symptom severity significantly predicted the ego's higher risk of having moderate-to-severe and severe symptoms of depression and anxiety. Our findings thus extend prior findings on the role of peers with severe symptoms in the transmission of mental health problems [6, 8–10, 16]. While previous studies in institutionally imposed group settings predominantly focused on the transmission associated with exposure to peers with severe symptoms [6, 16], our findings further showed that transmission can exist regardless of symptom severity of exposure. Our results for the exposure to classmates with mild-to-severe symptoms showed the effect sizes as IRR between 1.01 and 1.02. This indicated that a one-percentage increase of already affected classmates, regardless of symptom severity, corresponds to a 1%–2% higher risk of the ego reporting symptoms. This is of great importance considering the greater prevalence of students with mild-to-moderate mental health symptoms [28, 29]. Given the wide variety of exposure to already affected classmates with mild-to-severe symptoms (0%–59.0% for depression and 0%–65.7% for anxiety in our sample), a seemingly small effect size could imply a substantial change in students. Our results also further underscore the necessity of extending the estimate of interpersonal influence of mental health beyond close relationships. Nevertheless, our results cannot be directly compared with those from close relationships, such as friends. While previous studies investigating the transmission of depressive symptoms among friends reported a standardized β coefficient of 0.09 [8], this cannot be directly compared with our results from institutionally imposed groups due to potential reflection problems [39]. Specifically, individuals with mental health problems or vulnerabilities often self-select into peer groups with similar conditions [17]. Therefore, future studies are necessary to disentangle self-selection effects to enable comparisons with institutionally imposed group dynamics.

Moreover, benefiting from a complete dataset from entire classes, our study is the first to precisely estimate the level of exposure to already affected peers through calculating classroom density of already affected students. This precise estimation allowed for the identification of influential and interactive roles of classroom density of already affected students and symptom severity in the transmission. While previous studies only used number of peers with mental health problems as a predictor [6, 16], the use of classroom density of already affected students more accurately represented the group level exposure, as individuals are simultaneously exposed to both already affected and healthy peers [16]. Our results showed that the ego's risk of worse symptoms is associated with exposure to already affected classmates regardless of their symptom severity only when considering the proportion of already affected classmates in the classroom. While supporting previous studies that found a dose-responsive relationship [6, 16], our findings further indicated that the dose-responsive relationship should be interpreted with consideration of group density rather than mere number of already affected peers. Conversely, exposure to classmates with severe depressive or anxiety symptoms was significantly associated with the ego's worse symptoms, regardless of the proportion of these classmates in class.

This interaction between classroom density of already affected peers and symptom severity also indicated potential differences in the mechanisms underlying transmission of mental health problems associated with exposure to peers with severe or mild-to-moderate symptoms. One potential difference in the underlying mechanisms might stem from the symptom structures of mental health problems, which vary with severity [42]. Severe depression and anxiety may present symptoms that are more potent for transmission compared to milder conditions. However, the role of symptoms structures still requires further investigations. Apart from symptoms structure, it can also be inferred that transmission associated with exposure to severe and mild-to-moderate symptoms might manifest through different pathways. Specifically, severe symptoms are more noticeable in peer groups than milder conditions [43], thus inducing the risk of direct exposure. This visibility may facilitate social learning of maladaptive coping strategies [44], and amplify emotional contagion through direct interpersonal paths such as inducing empathy [45], stimulating mirror neuron reactions in the originally unaffected students [7], or interpersonal behaviors such as co-rumination [8]. This is in line with our finding that exposure to classmates with severe symptoms is associated with the ego's mental health symptoms, regardless of classroom density of these classmates. Alternatively, the transmission associated with subtle mild-to-moderate symptoms might only manifest when incorporating indirect group level mechanisms, as classroom dynamics are subjective to collective influences from both already affected and healthy students [16, 20]. According to the ecological systems theory, adolescent mental health development is influenced by multi-layer exposures to surrounding environments [23]. Classroom density of already affected peers captures both the microsystem, involving potential direct interactions with already affected classmates, and the mesosystem, reflecting indirect exposure to group dynamics as a result of interactions between classmates [24]. Therefore, the role of already affected peers with mild-to-moderate symptoms can be captured when considering both microsystem and mesosystem through classroom density. This might explain our finding that exposure to classmates with mild-to-moderate symptoms associated with the ego's mental health symptoms only when considering classroom density of these classmates. Regarding classroom level dynamics, the stress generation theory of mental health indicated that individuals who have or are vulnerable to mental health problems are at higher risk of experiencing stressful events that are partly dependent on themselves, with interpersonal stress being a major component [46–48]. Consequently, students with more classmates experiencing mental health symptoms are also at higher risk of encountering and witnessing interpersonal stressful events within class, leading to worse perceived classroom dynamic. Furthermore, individuals with mental health symptoms are less available for providing additional support to others [49], potentially leading to a perceived reduction in peer support within the classroom. Therefore, future studies might explore classroom level factors that may explain the transmission of mental health problems. Moreover, the role of classroom density of already affected students in the transmission of mental health problems also indicated a need to establish prevention strategies targeting potential mental health-related classroom level factors [20].

Beyond direct transmission, our results also support a bidirectional cross-transmission of depression and anxiety. Exposure to classmates with severe depressive symptoms was associated with higher risks of reporting worse anxiety, and vice versa. This aligns with the view of mental health problems as an interconnected spectrum [50]. Our results also align with evidence suggesting the prevalent comorbidity of depression and anxiety [2], as symptoms of one can trigger symptoms of the other. Notably, the cross-transmission was more pronounced with severe symptoms. Similar to direct transmission results, the cross-transmission associated with exposure to severe symptoms remained consistent across different exposure estimations. This potentially supports the fundamental differences between types of mental health conditions [51], wherein different conditions might only begin to share symptomatology as their severity increases. Specifically, individuals with non-severe mental health conditions are potentially more likely to display disorder-specific symptoms, fostering depression- or anxiety-oriented group dynamics that may drive symptom-specific transmission at the group level. In contrast, individuals with severe symptoms often exhibit prominent functional maladaptation linked to a broader spectrum of mental health disorders [52], increasing the risk of cross-transmission through direct interpersonal pathways [24]. These potential differences might be further explored in future studies.

Our results emphasize the pivotal need to address peer exposure to mild-to-moderate symptoms, alongside severe symptoms, in adolescent mental health research and prevention. Pending more rigorous examination through causal inference approaches, schools could consider implementing comprehensive screening systems to identify students with mental health difficulties early, using this data to guide intervention and prevention efforts. For instance, schools can strategically manage classroom compositions to prevent excessive clustering of already affected students, thereby reducing the risk of symptom transmission while ensuring a balanced and supportive peer environment. More importantly, the transmission of mental health symptoms does not imply that affected students should be isolated. Instead, efforts should focus on reducing stigma and fostering inclusivity [53]. Screening results must remain confidential, accompanied by regular anti-stigma campaigns and psychoeducation to normalize mental health discussions, encourage help-seeking, and foster a supportive atmosphere. A positive school climate, addressing mesosystem influences, can act as a social buffer, mitigating symptom transmission and alleviating existing problems [20, 54]. Collaboration with families, mental health professionals, and community organizations can further ensure sustainable, holistic prevention and intervention strategies [55, 56].

### 4.1. Strengths and Limitations

To the best of our knowledge, the present study is the first to utilize complete data from entire classes of students to investigate transmission of mental health problems with consideration of mild-to-moderate. This comprehensive dataset allows for precise estimation of levels of exposure to classmates with mental health problems and the calculation of classroom mental health problem density. However, few limitations should be acknowledged when interpreting the results. Most importantly, our findings are based on cross-sectional data, precluding the establishment of causality. Therefore, longitudinal studies are still needed to further validate our results. Another issue stemmed from the cross-sectional nature of our data is that, to avoid potential bias, the participant groups involved were not identical between the analyses of the roles of exposure to classmates with severe symptoms and exposure to classmates with mild-to-severe symptoms. Therefore, our results might be influenced by the different participant groups. Although risks of having each level of symptom severity were assessed separately to improve comparability, further investigations with identical outcomes are still in need to more effectively assess the difference brought by incorporating mild-to-moderate symptoms as exposures in transmission. Another limitation is the observational nature of our study, which makes the findings susceptible to unobserved confounding effects from individual, environmental, and socioeconomic factors [57]. Our analyses only controlled for basic demographics information, leaving room for potential confounding factors such as socioeconomic status and social support outside the school environment [58, 59]. Given China's “nearby enrollment policy” for middle schools, the random effects controlled for at the classroom and school levels can potentially account for some socioeconomic variance as students from the same school are likely to come from the same residential area [60, 61]. Nevertheless, our findings should still be further replicated after controlling for more potential confounding factors. Considering potential residual confounding effect, future explorations might also utilize quasi-experimental approaches with participant matching techniques to better facilitate a causal understanding of the transmission of mental health problems at a group level [62]. Moreover, while our study involved classes with a large variety of class size, we could only perform sensitivity analyses with regular-sized classes due to limited number of classes in the small and large class groups. Therefore, future studies could specifically focus on the group level transmission in small and large classes to assess whether the phenomenon vary in special settings.

## 5. Conclusions

Utilizing a comprehensive data from entire classes of Chinese middle school students, our study revealed that depressive and anxiety symptoms can be transmitted among adolescents through exposure to peers with mental health symptoms, irrespective of severity. The transmission of depressive and anxiety symptoms occurs not only in the presence of classmates with severe symptoms but also when having classmates with any symptom severity reaches a critical density in the classroom. Moreover, our findings highlighted that, in group level mental health transmission, the level of exposure should be interpreted with consideration of group density rather than mere number of already affected peers. This approach could potentially facilitate more accurate identification of at risk classes and support the development of targeted prevention strategies, pending validation through future experimental and quasi-experimental studies. Future investigations are suggested to explore potential class-level mechanisms of transmission and prevention targets to mitigate the transmission of mental health problems in the school environment.

## Figures and Tables

**Table 1:** Descriptive statistics of participants' characteristics.

Characteristics	Overall (*N* = 19,058)
Sex assigned at birth	
Girls	9213 (48.3%)
Boys	9845 (51.7%)
Grade	
Grade 7	7383 (38.7%)
Grade 8	4510 (23.7%)
Grade 9	7165 (37.6%)
Class size	48.00 (13.00, 104.00)
Depressive symptoms	1.50 (1.00, 5.00)
Anxiety symptoms	1.50 (1.00, 5.00)
Depression severity	
Health	13,407 (70.3%)
Mild symptoms	3892 (20.4%)
Moderate symptoms	1355 (7.1%)
Severe symptoms	404 (2.1%)
Anxiety severity	
Health	12,594 (66.1%)
Mild	4026 (21.1%)
Moderate	1774 (9.3%)
Severe	664 (3.5%)
Exposures to classmates with severe depression	
Count (*n*)	1.00 (0, 7.00)
Percentage (%)	2.04 (0, 12.50)
Exposures to classmates with severe anxiety	
Count (*n*)	1.00 (0, 11.00)
Percentage (%)	2.63 (0, 18.60)
Exposures to classmates with mild-to-severe depression	
Count (*n*)	13.00 (0, 46.00)
Percentage (%)	29.60 (9.86)
Exposures to classmates with mild-to-severe anxiety	
Count (*n*)	15.90 (7.30)
Percentage (%)	33.90 (11.10)

*Note:* Mean (SD) was presented for normally distributed continuous variables; Median (range) was presented for non-normally distributed continuous variables.

**Table 2:** Exposure to classmate depression and anxiety associated with ego's anxiety and depression, respectively.

In students without severe depressive symptoms	Depression-to-anxiety transmission					
	Number of classmates with severe depressive symptoms (*n*)			Percentage of classmates with severe depressive symptoms (%)		
	IRR	95% CI	*p* value	IRR	95% CI	*p* value
Mild-to-severe anxiety symptoms	1.04	(1.02, 1.07)	<0.001	1.02	(1.01, 1.03)	<0.001
Moderate-to-severe anxiety symptoms	1.08	(1.04, 1.12)	<0.001	1.04	(1.02, 1.06)	<0.001
Severe anxiety symptoms	1.16	(1.12, 1.20)	<0.001	1.07	(1.05, 1.09)	<0.001

**In students with mild-to-moderate depressive symptoms**	**Number of classmates with mild-to-moderate depressive symptoms (*n*)**			**Percentage of classmates with mild-to-moderate depressive symptoms (%)**		
	**IRR**	**95% CI**	***p* value**	**IRR**	**95% CI**	***p* value**

Mild-to-severe anxiety symptoms	1.00	(1.00, 1.01)	0.366	1.00	(1.00, 1.00)	0.356
Moderate-to-severe anxiety symptoms	1.01	(1.00, 1.01)	0.122	1.00	(1.00, 1.01)	0.133
Severe anxiety symptoms	1.01	(1.00, 1.02)	0.095	1.01	(1.00, 1.01)	0.072

**In students without severe anxiety symptoms**	**Anxiety-to-depression transmission**					
	**Number of classmates with severe anxiety symptoms (*n*)**			**Percentage of classmates with severe anxiety symptoms (%)**		
	**IRR**	**95% CI**	***p* value**	**IRR**	**95% CI**	***p* value**

Mild-to-severe depressive symptoms	1.03	(1.01, 1.05)	<0.001	1.02	(1.01, 1.02)	0.001
Moderate-to-severe depressive symptoms	1.05	(1.01, 1.09)	0.008	1.03	(1.01, 1.04)	0.008
Severe depressive symptoms	1.12	(1.07, 1.17)	0.028	1.08	(1.02, 1.14)	0.005

**In students with mild-to-moderate anxiety symptoms**	**Number of classmates with mild-to-moderate anxiety symptoms (*n*)**			**Percentage of classmates with mild-to-moderate anxiety symptoms (%)**		
	**IRR**	**95% CI**	***p* value**	**IRR**	**95% CI**	***p* value**

Mild-to-severe depressive symptoms	1.00	(1.00, 1.00)	0.722	1.00	(1.00, 1.00)	0.585
Moderate-to-severe depressive symptoms	1.00	(1.00, 1.01)	0.266	1.00	(1.00, 1.01)	0.074
Severe depressive symptoms	1.01	(1.00, 1.03)	0.086	1.01	(1.00, 1.02)	0.020

## Data Availability

The data that support the findings of this study are available from the corresponding author upon reasonable request.

## Supporting Information

Supporting Information 1 Figure S1: Mental health risks associated with exposure to classmates with mild-to-severe mental health symptoms in regular sized classes.

Supporting Information 2 Figure S2: Mental health risks associated with exposure to classmates with severe mental health symptoms in regular sized classes.

Supporting Information 3 Table S1: Exposure to classmate depression and anxiety associated with ego's anxiety and depression, respectively in regular sized classes.
