# Supplementary material for: Assessing Peer Exposure at a Group Level: The Role of Mild-to-Moderate Symptoms in the Transmission of Mental Health Problems
Source: Depress Anxiety. 2025 Apr 21;2025:1787378. doi: 10.1155/da/1787378 (PMC12037253; doi:10.1155/da/1787378)
Supplement: Supporting Information 2 — Figure S2: Mental health risks associated with exposure to classmates with severe mental health symptoms in regular sized classes. [file 1787378.f2.docx]

# **Figure S2.** Mental health risks associated with exposure to classmates with severe mental health symptoms in regular sized classes.


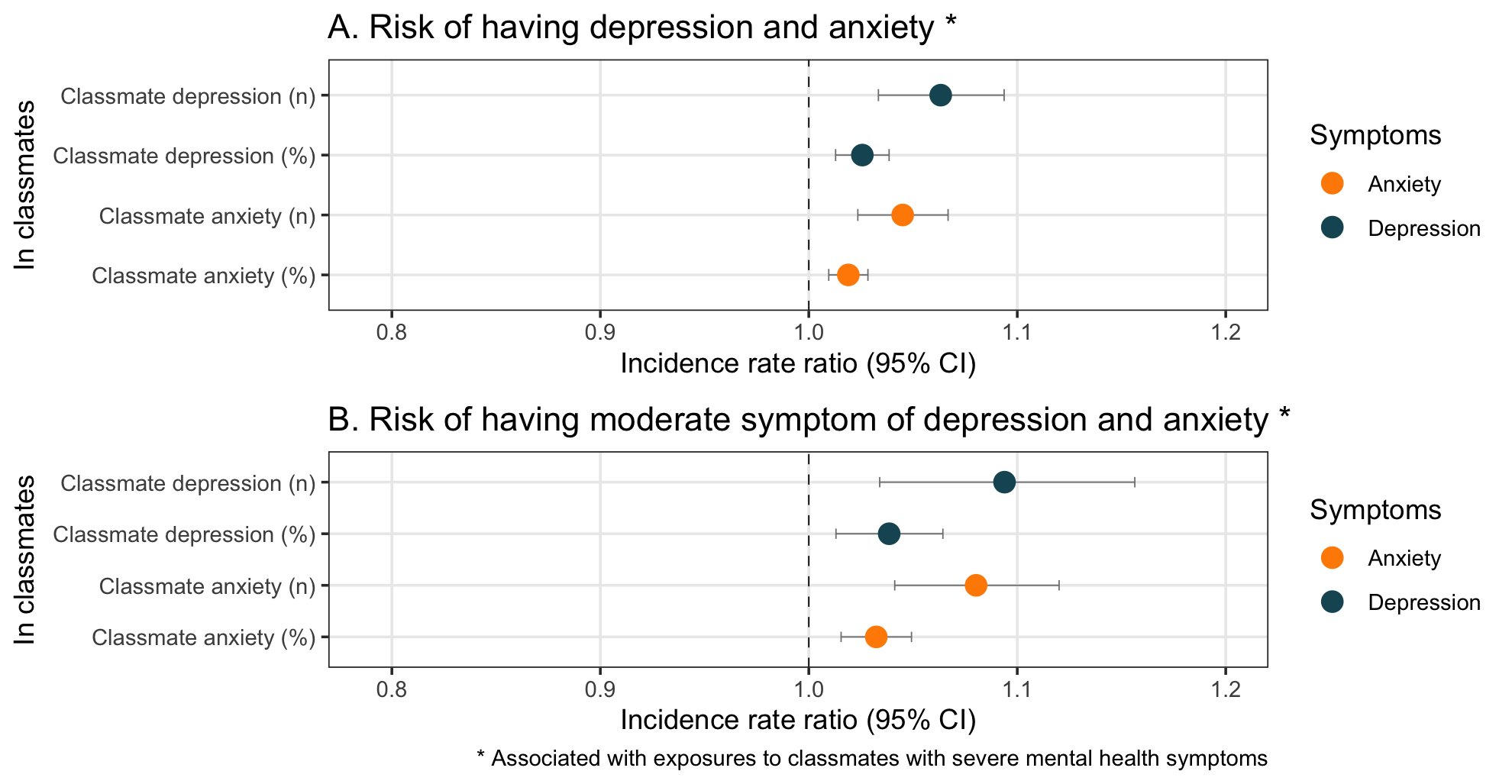


**Note:** In students with mild-to-severe symptoms. Random effects were controlled for at classroom and school levels.
