# Supplementary material for: Assessing Peer Exposure at a Group Level: The Role of Mild-to-Moderate Symptoms in the Transmission of Mental Health Problems
Source: Depress Anxiety. 2025 Apr 21;2025:1787378. doi: 10.1155/da/1787378 (PMC12037253; doi:10.1155/da/1787378)
Supplement: Supporting Information 3 — Table S1: Exposure to classmate depression and anxiety associated with ego's anxiety and depression, respectively in regular sized classes. [file 1787378.f3.docx]

| **Table S1.** Exposure to classmate depression and anxiety associated with ego’s anxiety and depression, respectively in regular sized classes. | | | | | | | |
| --- | --- | --- | --- | --- | --- | --- | --- |
|  | **Depression-to-anxiety transmission** | | | | | | |
| **In students without severe depressive symptoms** | **Number of classmates with severe depressive symptoms (n)** | | |  | **Percentage of classmates with severe depressive symptoms (%)** | | |
|  | **IRR** | **95% CI** | ***p*-value** |  | **IRR** | **95% CI** | ***p*-value** |
| Mild-to-severe anxiety symptoms | 1.06 | [1.03, 1.09] | <0.001 |  | 1.02 | [1.01, 1.04] | <0.001 |
| Moderate-to-severe anxiety symptoms | 1.10 | [1.05, 1.15] | <0.001 |  | 1.04 | [1.02, 1.06] | <0.001 |
| Severe anxiety symptoms | 1.18 | [1.06, 1.30] | 0.002 |  | 1.07 | [1.03, 1.12] | 0.001 |
| **In students with mild-to-moderate depressive symptoms** | **Number of classmates with mild-to-moderate depressive symptoms (n)** | | |  | **Percentage of classmates with mild-to-moderate depressive symptoms (%)** | | |
|  | **IRR** | **95% CI** | ***p*-value** |  | **IRR** | **95% CI** | ***p*-value** |
| Mild-to-severe anxiety symptoms | 1.00 | [1.00, 1.01] | 0.414 |  | 1.00 | [1.00, 1.00] | 0.406 |
| Moderate-to-severe anxiety symptoms | 1.01 | [1.00, 1.02] | 0.111 |  | 1.00 | [1.00, 1.01] | 0.158 |
| Severe anxiety symptoms | 1.02 | [1.00, 1.04] | 0.123 |  | 1.01 | [1.00, 1.02] | 0.122 |
|  | **Anxiety-to-depression transmission** | | | | | | |
| **In students without severe anxiety symptoms** | **Number of classmates with severe anxiety symptoms (n)** | | |  | **Percentage of classmates with severe anxiety symptoms (%)** | | |
|  | **IRR** | **95% CI** | ***p*-value** |  | **IRR** | **95% CI** | ***p*-value** |
| Mild-to-severe depressive symptoms | 1.04 | [1.02, 1.06] | <0.001 |  | 1.02 | [1.01, 1.03] | 0.002 |
| Moderate-to-severe depressive symptoms | 1.06 | [1.01, 1.11] | 0.022 |  | 1.02 | [1.00, 1.05] | 0.019 |
| Severe depressive symptoms | 1.21 | [1.06, 1.38] | 0.003 |  | 1.10 | [1.03, 1.16] | 0.001 |
| **In students with mild-to-moderate anxiety symptoms** | **Number of classmates with mild-to-moderate anxiety symptoms (n)** | | |  | **Percentage of classmates with mild-to-moderate anxiety symptoms (%)** | | |
|  | **IRR** | **95% CI** | ***p*-value** |  | **IRR** | **95% CI** | ***p*-value** |
| Mild-to-severe depressive symptoms | 1.00 | [1.00, 1.01] | 0.583 |  | 1.00 | [1.00, 1.00] | 0.516 |
| Moderate-to-severe depressive symptoms | 1.01 | [1.00, 1.02] | 0.275 |  | 1.00 | [1.00, 1.01] | 0.076 |
| Severe depressive symptoms | 1.03 | [1.00, 1.05] | 0.033 |  | 1.01 | [1.00, 1.02] | 0.025 |
